# Sequence evidence for common ancestry of eukaryotic endomembrane coatomers

**DOI:** 10.1038/srep22311

**Published:** 2016-03-02

**Authors:** Vasilis J. Promponas, Katerina R. Katsani, Benjamin J. Blencowe, Christos A. Ouzounis

**Affiliations:** 1 Bioinformatics Research Laboratory, Department of Biological Sciences, New Campus, University of Cyprus, PO Box 20537, CY-1678 Nicosia, Cyprus; 2 Department of Molecular Biology & Genetics, Democritus University of Thrace, GR-68100 Alexandroupolis, Greece; 3 Donnelly Centre for Cellular & Biomolecular Research, University of Toronto, 160 College Street, Toronto, Ontario M5S 3E1, Canada; 4 Biological Computation & Process Laboratory (BCPL), Chemical Process Research Institute (CPERI), Centre for Research & Technology (CERTH), PO Box 361, GR-57001 Thessalonica, Greece

## Abstract

Eukaryotic cells are defined by compartments through which the trafficking of macromolecules is mediated by large complexes, such as the nuclear pore, transport vesicles and intraflagellar transport. The assembly and maintenance of these complexes is facilitated by endomembrane coatomers, long suspected to be divergently related on the basis of structural and more recently phylogenomic analysis. By performing supervised walks in sequence space across coatomer superfamilies, we uncover subtle sequence patterns that have remained elusive to date, ultimately unifying eukaryotic coatomers by divergent evolution. The conserved residues shared by 3,502 endomembrane coatomer components are mapped onto the solenoid superhelix of nucleoporin and COPII protein structures, thus determining the invariant elements of coatomer architecture. This ancient structural motif can be considered as a universal signature connecting eukaryotic coatomers involved in multiple cellular processes across cell physiology and human disease.

Nuclear pore complexes (NPCs) are modular assemblies embedded at the points of fusion between the inner and outer membrane of the eukaryotic nucleus that mediate nucleocytoplasmic transport^1^. The overall architecture and composition of the NPCs is largely taxonomically conserved, indicating early origins in the eukaryotic tree^2^. In particular, nucleoporins including those at the outer ring coat forming the Y-complex (outer ring coat Nups or Y-Nups) share certain key structural and architectural similarities, possibly due to deep divergence^3^,^4^,^5^. These features extend beyond the nuclear pore, namely the COPII coat associated with anterograde transport from the rough endoplasmic reticulum to the Golgi apparatus and the COPI coat associated with the reverse, retrograde transport^6^, suggesting a common origin of endomembrane coatomers, one class of which is represented by the NPC coat^7^.

The divergence of nucleoporin families has been proposed on the basis of global structural but no specific sequence evidence, especially for the Y-Nups^8^. This presumption is based on detailed structural analysis, presence of beta-propeller repeats at the N-terminus, an alpha-solenoid superhelix at the C-terminus and other architectural elements with regard to the multi-domain composition of Y-Nups^5^,^9^. In particular, the solenoid superhelix of the resolved structures for Nup75, Nup96, Nup107 (Y-Nups) and Nic96 – reminiscent of the tetratrico-peptide repeat (TPR) domain^10^ – is also present in Sec31 and Sec16, building blocks of the COPII vesicle coat^11^,^12^. Much attention has been paid to this structural element as a common architectural motif across diverse coatomer molecules, and has thus been named Ancestral Coatomer Element 1 (ACE1)^11^,^13^, favouring the hypothesis of deep divergence over convergent evolution^14^. Yet, no sequence signature for ACE1 has ever been detected, either for Y-Nups/Nic96 or Sec31/Sec16, while the structure determination and comparison of these coatomers revealed this surprising structural similarity^11^. ACE1 might be considered as a structural manifestation of the likely common origin of NPC and COPII coats^13^, but has never been observed outside these complexes^15^. A combination of phylogenomic profiling and structural predictions has further extended this relationship to the intraflagellar transport complex (IFT) of the cilium^16^, across eukaryotic phyla and their representative genome sequences^17^. Affirming an earlier hypothesis for the homology of the IFT complex with endomembrane coatomers^18^, IFT-A components IFT122, IFT144/WDR19 and WDR35 and IFT-B components IFT172 and IFT80 are detected as ancestrally related to COPI subunits, yet without a connection to nucleoporins or a reference to the ACE1 structural motif^17^. Therefore, despite abundant sequence and structural data for this motif, the identification of ACE1-containing molecules and their relatives remains highly challenging, a task partly achieved only by a mixture of sequence profiles, alpha helical predictions and domain architecture considerations^11^. Herein, we present sequence evidence for the long suspected common origin of NPCs, COPIIs, IFTs and other coatomer systems across eukaryotes, unifying previous insightful hypotheses and detailed structural studies^19^.

## Results

**Figure 1:**
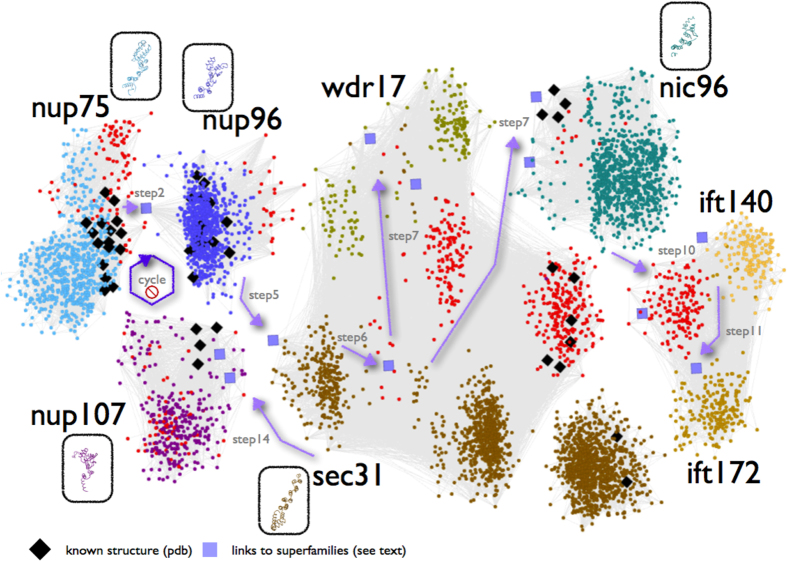
Pictorial representation of the sequence space walk connecting components of the nuclear pore complex, COPII and intraflagellar transport. Members of each of the eight superfamilies are shown in distinct colors, with superfamily representatives of known structure enclosed in oval boxes following the same coloring scheme. Orientations of structural representatives are identical to the reference Nup75 structure (3F3F_C, Figure 2a). Two exceptions of the coloring scheme involve homologs of known structure (black diamonds-♦) and previously uncharacterized (unannotated) protein sequences (red dots-●). Intra-family connections detectable by pairwise sequence comparisons are represented by dense sub-networks of thin light-grey lines (see Methods). Inter-family connections revealed by iterative profile searches – otherwise undetectable, are depicted by (i) light purple arrows for the corresponding steps and (ii) squares for sequence links across superfamilies. Seven steps of the sequence space walk deemed as critical for revealing novel inter-family relationships are displayed (step 7 is shown twice, as it connects both to WDR17 and Nic96 superfamilies) – see also Table S2. The cycle with a ‘stop’ sign refers to the exclusion of Nup107 superfamily which terminates the search early (see Methods). Only a representative subset of hits is shown for clarity; this representation should only be taken as a rough sketch of the documented process (available as Data Supplement DS05). An annotated version of this two-dimensional layout is available as Data Supplement DS11.

In our quest for multi-domain architectures for the structural and functional analysis of the Y-complex^20^, we have encountered a unique, subtle sequence similarity with a critical, missing link between the Nup75 sequence profile and the Nup98-96 (Nup96) sequence of the insect species *Harpegnathos saltator* (GI:307191801)^21^. Thanks to the recent availability of genomic information across many eukaryotic genomes, gaps in this particular region of genome sequence space are being filled rapidly by homologs which can connect hitherto seemingly unrelated protein sequence families – in this case Y-Nups, via significant sequence similarities (see also Supplementary Text). We have further pursued a rigorous analysis of this puzzling connection between Nup75 and Nup96 superfamilies by conditional iterative sequence profile searches, using the Nup75 sequence profile as a query – Nup75 alignment positions 339-2024 in DS03 of our previous report^20^ (Data Supplements DS01-DS04); profiles are represented as position-specific scoring matrices (PSSMs). By inspecting thousands of alignments, we were able to detect sequence signals of the divergent alpha-solenoid superhelix within 3,502 sequences in the non-redundant protein database (effective date December 2013) (Figure S1). Having initially excluded Nup107 (which terminates the search early, see Methods), we uncover the deeply divergent sequence relationships between Nup96, Sec31, WDR17, Nic96, IFT140 (from IFT-A), IFT172 (from IFT-B) and finally Nup107 in this order (Fig. 1) – while IFT144/WDR19 and IFT122 (IFT-A components), Sec16 and Clathrin (but not COPI) are marginally detected beyond the set threshold, thus unifying NPC, IFT, COPII, and Clathrin^22^,^23^, as well as uncharacterized molecules such as WDR17 (see Supplementary Text).

This complex sequence profile search has been crucially based on the manual exclusion of 15 putative false positive cases (and, hence, of their homologs in future searches) (Table S1), some of which tend to appear in our profile sequence queries more than once. At each step, this procedure – which can be regarded as a genuine *sequence space walk* – unravels specific subsets of increasingly distant homologs in a highly controlled manner (Figure S1, Table S2). To ensure reproducibility, we have carefully repeated and documented these profile searches, until the process encounters noise, i.e. spurious sequence similarities for which no evidence of ACE1-containing motifs is available either in annotation records or reverse sequence searches (Data Supplement DS05). A visual representation of an increasingly sensitive sequence profile across sequence space is provided as a video file (Video S1). To assess coverage, we have also interrogated the protein database using Entrez^®^ text queries, and retrieved 5662 redundant entries (61 duplicate, 5601 unique), many of which, however, represent false positive identifications of the corresponding motifs (by automatic assignment) (Data Supplement DS06). To maintain precision at virtually 100% (Figure S1), as indicated by detailed structural validation and interpretation (see below), coverage is somewhat compromised for this particular database search and is indeed under-estimated: in principle coverage can be increased by imposing length constraints along the text query, similarly to sequence searches. The iteratively derived profile named KMAP-13 for ‘euKaryotic endoMembrane ACE1 Profile at Step 13’ is made available (Data Supplement DS07), along with the hit table containing sequence identifiers, to facilitate the extraction of the corresponding database entries and future updates (Data Supplement DS08).

**Figure 2:**
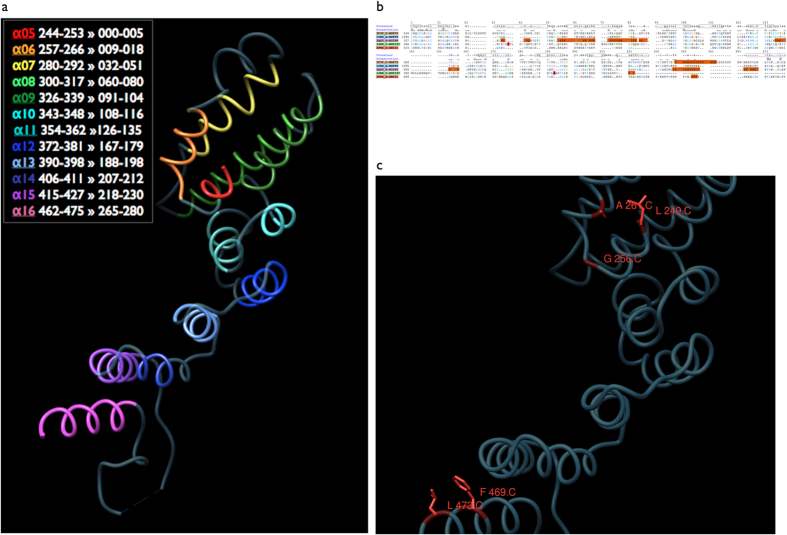
Sequence conservation across endomembrane coatomer structure components. (**a**) The Nup75 structure (3F3F_C) corresponding to the detected ACE1-like alpha-solenoid superhelix motif is shown. Individual helices α5-α16 are colored by unique colors, warm colors representing the most conserved segments (α5-α6, α15-α16). Sequence positions for each helix are shown in the legend, according to the comparative structural analysis of ACE1^11^, followed by the corresponding positions of the alignment in Fig. 2b. This reference orientation is used throughout this work, corresponding to the crown and the second half of the ACE1 trunk – see also Fig. 5 in Ref. 11. (**b**) Sequence alignment of five alpha-solenoid superhelix motif-containing representative structures. Aligned positions are established by direct comparison of the KMAP-13 profile against the structure database, edited to match the reference Nup75 structure, using Mview^44^. PDB codes are given, followed by the description of the corresponding protein chains. A consensus sequence and a skyline conservation plot are provided. The twelve helices listed in Fig. 2a are depicted as horizontal oval-shaped bars marked for the consensus sequence, seven at the top panel and the remaining five at the bottom panel. The total length of the alignment is 280 residues, corresponding to the KMAP-13 profile search against the common conserved elements of the available structures^11^. Regions missing in the structure database entries are shown in orange. Structural data in PDB format are provided in Data Supplement DS09. (**c**) Structural context of the evolutionarily conserved positions in Nup75. N-terminal Leu-249, Gly-256 and Ala-261 (C for chain C of 3F3F) (see Fig. 2a) and C-terminal Phe-469 and Leu-473 correspond to alignment positions I1, G8, A13 and F272, L278 respectively (Fig. 2b). The C-alpha trace is shown in dark blue and the subset of conserved positions is shown in red, along with the side chain representations. Alignment position L101 is not highlighted as it is frequently substituted by a tyrosine residue (Fig. 2b).

A key result of this sequence space exploration is the demonstration that three nucleoporin superfamilies – namely Nup75, Nup96, Nup107 – not only share structural similarities but these similarities arise from divergent evolution at very low sequence identity levels (minimum sequence identity across runs 3–9%, average 6.8%) with statistically-significant alignments (p < 0.001). Our sequence profile searches unambiguously underline the deep phylogenetic connection of these Y-Nups, as well as Nic96 and Sec31 (see Supplementary Text). In this well-defined, newly discovered sequence space locality of these homologous molecules containing the alpha-solenoid superhelix, there are five protein superfamilies represented by resolved three-dimensional structure homologs, namely Nup75, Nup96, Nup107, Nic96 and Sec31 (Fig. 1) – the corresponding Sec16 region is also correctly detected, albeit below threshold. To validate the sequence profile-driven alignments, we superimposed the four known nucleoporin as well as Sec31-COPII structures on the basis of aligned positions for five conserved residues: the structural superposition verifies our results, as four structures are superimposed precisely along the alpha-solenoid superhelix with RMSD values <3 Å for C-alpha atoms (Figure S2, Figure S3 – except Sec31’s last helix hairpin) – with better fit towards the N-terminal part of the ACE1 alpha-solenoid. Surprisingly, this is the first time that sequence information alone strongly reflects the structural similarity of these molecules as previously observed^13^, thus both delineating the evolutionary history of ACE1-like motifs and supporting the hypothesis that coatomer systems arose by divergent evolution^24^ (Fig. 2). Furthermore, the structural partitioning of ACE1 and relatives into crown (α5–11), trunk (α1–3,α13–19) and tail (α21–28) can now be viewed from an evolutionary perspective, where helices α5-16 across the crown and trunk segments represent the conserved core of ACE1 (Fig. 2a). In a length of 280 residues, there are only three invariant positions linked by divergence: Ala (A13), Phe (F272), Leu (L278) (Fig. 2b); a number of other conserved positions are also observed namely Ile (I1), Gly (G8), Tyr (Y101) – both these sets are used for structural superposition. It should be noted that the full alignment unravels limited variation across these positions, for example A13 is 84% present sporadically substituted by Ser or Thr and F272 is frequently substituted by Tyr (enumerated by profile alignments across significant sequence similarities in database searches, Data Supplement DS05). In all, our results show that the region corresponding to helices α5–16 represents the only common structural element across the five coatomer structures detectable at the sequence level in the available structure coordinate data, while linker domains within this region modify relative orientations, as previously indicated^11^. This, in turn, hinders detection of additional ACE1 proteins^11^ and renders the identification of common elements at the sequence level crucial, yet highly challenging.

The deep connections brought to light by this sequence space walk resolve a long-standing issue of coatomer phylogeny across eukaryotes and permit the evolutionary dissection of rich structural data for ACE1 alpha-solenoids (Fig. 2). Remarkably, helix α8 of Nup75^13^, corresponding to helix α7 of ACE1^11^, does not exhibit conservation across those families as previously reported^13^. The most conserved block of ACE1 resides towards the N-terminal part of the crown, namely helices α5-α6 (positions 1-22, Fig. 2b,c). The alignment quality decreases towards the C-terminal part, with the exception of invariant positions 272 and 278 (undetectable in the Nup107 structure – 3jroC). The conserved ACE1 block ranges between invariant alignment positions I1 (α5 position 11; Ile in other structures, Leu-249 in Nup75) and A13 (Ala-261) maintaining the hairpin α5-α6 contact (Fig. 2c), while position G8 (Gly-256, Asn in Nup96) acts as helix breaker for α5. At the C-terminal region of the conserved ACE1 block, position F272 (Phe-469) packs against position L278 (Leu-473), stabilizing α16 with α15 of the trunk, and possibly α1 as well (Fig. 2c). We reason that these non-polar residues might not contribute towards interface contacts and are most likely involved in maintaining the ancestral structural integrity of ACE1, despite astonishing variation acquired elsewhere in this structural motif^25^. This is a testable prediction that could be validated by assessing the impact of evolutionarily conserved regions^20^ for the stability of endomembrane coatomer components represented by the currently available structures as above.

## Discussion

The evolutionary dissection of endomembrane coatomers exhibits a strong structural conservation of the alpha-solenoid superhelix with family-specific sequence variation and adaptive association with beta-propeller motifs in the case of the Y-complex, e.g. Nup75 with Seh1 and Nup96 with Sec13^9^. A particular instance of the coatomer superhelix shared between nucleoporins and COPII components has been attentively termed ACE1 based on structural similarities^13^. Our analysis provides specific sequence evidence for the deep divergence of three Y-Nups/Nic96 and COPII, as previously proposed on structural grounds^19^. We further extend the presence of the coatomer superhelix to some of the longest components of the IFT, namely IFT140 and IFT172, recently predicted as remote relatives by phylogenomic analysis^17^. The detection of a number of IFT core components^26^ by this sequence space walk suggests that they play a key role in the ciliary pore complex (CPC) that regulates transport^27^, analogously to the NPC^28^. Further structural analysis of IFT components, so far achieved for a number of smaller IFT core proteins^29^, can unravel the alpha-solenoid superhelix in some of the longest IFT members, such as IFT144 or IFT172, involved in human skeletal ciliopathies^30^. The identification of WDR17 points to its involvement in eye gene expression and possibly disease^31^, further supported by positive selection pressure in dolphin sensory systems^32^ and by proteomics detection as a conserved element in Joubert Syndrome-associated ciliary signaling subdomain ARL-13^33^. The compelling sequence similarity across coat complexes and associated processes enhances proposals about a common origin of endomembrane coatomers early in eukaryotic evolution^34^,^35^. The puzzling connections revealed by deep divergence of coatomers offer new perspectives for their emerging implication in coupling multiple cellular roles, such as the kinetochore involving the Y-complex^36^, nucleoporins associating with histone-modifying complexes^37^ and the centrosome connecting to the nucleus, the Golgi apparatus and the eukaryotic cilium^38^.

## Methods

### Sequence comparison

Sequence entries were obtained from the NRDB database, available at the NCBI^39^. Sequences were filtered by CAST^40^, and searches were performed by PSI-BLAST^41^, against NRDB. The conditional iterative profile sequence searches were set with default values, excluding compositional based adjustments (replaced by CAST for compositional bias masking), and the following parameters: search was performed against eukaryotes only, target sequence length range was set to 300-10000 residues to exclude fragments and spurious hits against short proteins, Nup107 was excluded for the 13 iterations and admitted only at iteration 14, e-value threshold was set to 10^−03^, maximum number of targets was increased to 20,000, expectation threshold was set to 1.

The key symbolic parameter, namely the exclusion of Nup107 annotation entries (domain name Nup84_Nup100, Pfam identifier PF04121), is set to disregard Nup107 superfamily entries, which otherwise accelerate early convergence, representing over-training of the sequence space walk assessed by a high number of false negative cases (see below, Validation and clustering). This parameter is expressed in Entrez^®^ query, as follows: “NOT Nup84_Nup100[All Fields]”. It is remarkable that despite the purposeful exclusion of Pfam-annotated Nup107 entries, the sequence space walk returns certain members of this superfamily (see Supplementary Text and Table S2), at step 8 onwards. The profile search matches those un-annotated entries at exactly the same region reported for their annotated (excluded) counterparts, indicating a deep evolutionary connection (not shown; example: Step 8 in Data Supplement DS05 XP_005397534.1 matches profile correctly at positions 256-596). This provides additional, strong evidence that the profile search maintains key sequence features of eukaryotic coatomer superfamilies, including Nup107s, which are not necessarily annotated in the database.

False positives were assigned on the basis of reverse BLASTp searches against NRDB. All alignments were manually inspected to exclude those potential false positive cases, deemed as such by individual database searches, absence of relevant sequence motifs in the corresponding database records or any other kind of supporting evidence. It should be noted that the exclusion of possible true positives marked as false positives (2 instances, Table S1), does not impact the ultimate result. We have performed this analysis with various starting points and provide the most accessible instance, based on previous work and multiple repetitions of the process (not shown), until reproducibility was established. In fact, in other incarnations of the profile search without excluding Nup107s and various significance thresholds (see Supplementary Text), cross-superfamily relationships are still detected – yet, without sufficient coverage, i.e. without detecting previously annotated database entries belonging to any of the target superfamilies.

### Structure comparison

Profile-driven alignments were generated from KMAP-13 against the PDB database, and the corresponding entries were edited to correspond to the final alignment (as in Fig. 2b). These structure coordinate files are also provided as Data Supplement DS09 in ZIP format. Structure visualization and interactive analysis was supported by Chimera^42^ – alignments follow the Clustal X color scheme. Additional structure comparisons were performed by DALI/FSSP^43^. Conservation was assessed by structural superposition dictated by the invariant positions of the sequence alignment.

Multiple alignment construction from BLAST searches was facilitated by Mview^44^. Results of the structural alignment confirm the sequence profile-driven alignments with slight, single-residue mismatches or gap variations (Data Supplement DS10, filtered by the following criteria: alignment length >125, rmsd <10 Å; seqidentity: >8%). Putative false positives of the DALI search are kinesin light chain 1 (3nf1A) and cytoplasmic export protein 1 (3vwaA), both containing alpha-helical segments.

### Validation and clustering

To assess coverage of the discovered ACE1-containing proteins based on the sequence space walk, we deployed the following Entrez^®^ query which returns putative members of our target superfamilies based on manual or computationally inferred annotations included in all text fields as below.

Nucleopor_Nup85[All Fields] OR Nup96[All Fields] OR Nup107[All Fields] OR Nic96[All Fields] OR (“intraflagellar transport protein 140”[All Fields] OR ift140[All Fields]) OR (“intraflagellar transport protein 172”[All Fields] OR ift172[All Fields]) OR (“WD repeat-containing protein 17”[All Fields] OR WDR17[All Fields]) OR ACE1-Sec16- like[All Fields].

This query returns 5662 protein sequence entries which are further clustered to assess internal consistency based on sequence cross-similarities, using CAST for filtering (threshold ≥20) and BLAST (e-value ≤10^−06^) for matching. From a database annotation standpoint, it is worth noting that the above target superfamilies are currently described by at least ten different profiles in Pfam, not necessarily connected between them. Sequence clustering of BLAST similarities was performed by TribeMCL^45^ (inflation value = 2.0) and sequence relationships were visualized using BioLayout^46^. Similarly, our recovered 3,502 homologs were also clustered in the same mixture (selected for <25% sequence identity), with same parameters – select group assignments are provided in Data Supplement DS11, in BioLayout format.

Redundant sequence entries extracted by annotation as above are 5601 (3634 common +1967 unique by annotation), while sequence entries extracted by profile sequence searches are 4414 (3634 common +780 unique by sequence). Non-redundant entries are 3017 and 533 for annotation and sequence respectively, and shared entries in this set are 2584 detected by profile searches and also supported by the corresponding annotations.

Our conditional iterative profile sequence search strategy using various filters for both sequence features (e.g. bias, length) and annotation records (e.g. description, taxonomy) represents a novel, general approach that can be modified to delineate complex cases of enigmatic superfamily relationships.

## Additional Information

**How to cite this article**: Promponas, V. J. *et al.* Sequence evidence for common ancestry of eukaryotic endomembrane coatomers. *Sci. Rep.* **6**, 22311; doi: 10.1038/srep22311 (2016).

## Supplementary Material

Supplementary Information

Supplementary Video S1
