# Supplementary material for: Sequence evidence for common ancestry of eukaryotic endomembrane coatomers
Source: Sci Rep. 2016 Mar 2;6:22311. doi: 10.1038/srep22311 (PMC4773986; doi:10.1038/srep22311)
Supplement: Supplementary Information [file srep22311-s1.pdf]

Sequence evidence for common ancestry of eukaryotic endomembrane coatomers

2015|11|20

Vasilis J. Promponas[1], Katerina R. Katsani[2], Benjamin J. Blencowe[3], Christos A. Ouzounis[1,3,4*]

[1] Bioinformatics Research Laboratory, Department of Biological Sciences, New Campus, University of Cyprus, PO Box 20537, CY-1678 Nicosia, Cyprus

[2] Department of Molecular Biology & Genetics, Democritus University of Thrace, GR-68100 Alexandroupolis, Greece

[3] Donnelly Centre for Cellular & Biomolecular Research, University of Toronto, 160 College Street, Toronto, Ontario M5S 3E1, Canada

[4§] Biological Computation & Process Laboratory (BCPL), Chemical Process Research Institute (CPERI), Centre for Research & Technology (CERTH), PO Box 361, GR-57001 Thessalonica, Greece

[*]corresponding author: CAO, email: ouzounis@certh.gr {[§]present address}

Supplementary Information
Supplementary Text
Figures S1 to S3 (3)
Video S1 (1)
Tables S1 to S2 (2)
Data Supplements DS01 to DS11 (11)
References (47–63)

SUPPLEMENTARY INFORMATION

Supplementary Text

The molecular architecture of the nuclear pore coat [5] shares common elements with the COPII coat involved in anterograde vesicular transport and the COPI coat involved in the retrograde transport [47]. The sequence space landscape of these eukaryotic coatomers is very complex reflecting their evolutionary histories [48], confounded by low sequence identity across protein superfamilies (e.g. Nup107, Nic96, Sec31) [49] and noisy next-generation sequencing data. While our discovery has been initially based on serendipitous observations, we hypothesized the conservation of the central core of the ACE1 motif, and have been systematically devising schemes to explore this landscape by profile searches and clustering protocols. As we have previously demonstrated [20], the seven major superfamilies of the nine Y-Nups (one superfamily covering all Sec13/Seh1/Nup37 members) are additionally accompanied by two smaller sub-families, namely Nup75 and Nup107. These sub-families include some of the most distant nucleoporin sequences made available from genome projects, representing missing links that can be exploited to link families together [50]. Starting from a number of seed sequences, e.g. Nup75 and Nup96, we have performed profile-driven PSI-Blast iterative searches [41], characterizing the maximal possible extent of these two Y-Nup superfamilies which subsequently define features that capture the ACE1 motif, with methods for low-complexity masking [40] and variable significance thresholds [20].

These similarities can indeed be established by an arbitrary number of starting points in this sequence space locality; consequently, we have distilled the essence of this discovery by establishing a reproducible and highly controlled profile search – with a sequence profile library called KMAP (euKaryotic endoMembrane ACE1 Profile) – as a walk in sequence space (Figure 1), annotated at each step (Table S2). The statistical significance threshold has been heuristically set for the given input profile from Nup75 – itself produced by 4 iterations in previous work [20]: 10^{-03} produces an optimal result reported herein; threshold value 10^{-02} is too sensitive (too many false positive cases), while threshold value 10^{-04} is too specific (requiring 72 iterations to reach a similar amount of significant alignments – not shown).

The query profile was enriched by another 14 iterations, a total of 18 iterations, capturing the discovered similarities into a reproducible and calibrated sequence search scheme, which can be considered as a walk in sequence space [50]. This process can be depicted as an iterative sequence profile computation, with high specificity and good sensitivity (Figure S1). This accurate profile, called KMAP-13 (Data Supplement DS07), at iteration 13 is able to capture 3502 homologs, with the exclusion of very few putative false positive cases (Table S1). Each iteration yields on average 269 new members (standard deviation 220) matching the sequence profile (ranging between 15 at iteration 2 and 659 at iteration 7). All resulting data, including alignments as well as the technical details are provided, to ensure reproducibility and support further discovery. The detected similarities are of profound significance, as they finally resolve the proposed hypothesis of divergent evolution of coat nucleoporins, previously only alluded to by structural superposition of folding motifs and common architectural elements [5,9].

We describe the traversal of the sequence space starting with Nup75 as a profile query [20], itself generated by four iterations (Table S2). All results are provided in various formats from the PSI-BLAST runs (Data Supplement DS05). At step 1, more Nup75 members are detected, while at step 2, the first members of Nup96 homologs, primarily from insects emerge as significant hits. At step 3, the two superfamilies Nup75 and Nup96 merge into a large group of

homologous proteins, matching the ACE1-equivalent segment, returning the third most numerous set of 'new' hits. At step 4, only a few additional hits are found at the required significance level: this step represents a bottleneck that is subsequently surpassed. At step 5, more Nup96 members are detected along a Sec16-like ACE1-containing molecule from *Branchiostoma floridae* (GI:260816342). At step 6, remarkably, significant hits are enriched in yet more Nup96 homologs with the appearance of Sec31A members. At step 7, the search yields the most numerous set of 'new' hits, including a multitude of Sec31 homologs, as well as a few Nic96 members and a handful of WDR17 poorly characterized proteins [51]. At step 8, new hits are primarily represented by WDR17's, some Sec31A and Sec31B homologs, a few unannotated Nup107s, and the first IFT140 member, again from *Harpegnathos saltator* (GI:307206999). At step 9, the second most numerous set of 'new' hits is generated, covering a multitude of homologs from a wider range, including primarily Nic96 members, IFT140 members, and Sec31 subunits. At step 10, multiple entries belonging to previously found families are detected, as well as a large group of IFT140 homologs, including many uncharacterized members of this superfamily – surpassing the 3000 homolog milestone, it is quite surprising that there are very few (if any) false positives above threshold at this point. At step 11, the detection of ACE1-like regions is consolidated across superfamilies, with the detection of the first members of the IFT172 superfamily, e.g. a 1735-residue long protein from *Camponotus floridanus* (GI:307182081) – interestingly, this species is represented by one member per family detected here at this stage (step 11). At step 12, new findings start to drop in relation to previous steps, and a certain consolidation is taking place, with more remote members of all previous superfamilies, especially non-nucleoporins, being detected. At step 13, a similar situation prevails, with the exception of a most remarkable hit, the Sec31 homolog of known structure (Figure 2b). At step 14, annotated homologs of Nup107 (i.e. detected by domain databases) are admitted, and the entire sequence space locality is sufficiently covered. It should be noted that the Nup107 structure is not detected by the process – it is added manually into the alignment by profile matching against the PDB (Figure 2b). A number of other interesting, marginally non-significant, hits include IFT-A components WDR19/IFT144 (e.g. *H. saltator*, GI:307199281) and IFT122 (e.g. *Micromonas pusilla* CCMP1545, GI:303272293); Sec16 is found once (*Wallemia ichthyophaga* EXF-994, GI:505759425) at positions 323-785, matching the structure with PDB code 3mzkB 15-441 and by extension 2pm6A at positions 150-280, consistent with the alignment; Clathrin heavy chain from *Schizosaccharomyces pombe* 972h- is also detected (GI:19115060), at positions 735-1186, indicating a remote profile affinity with the clathrin heavy chain repeat region. These tantalizing hits are detected correctly below threshold, as verified by reverse searches of the corresponding regions, thus closing the gap of the sequence space locality with Y-Nups [20], Nic96 [1,52], Sec31 [53] and IFT140/IFT172 [26].

Thus, the elusive sequence signature that connects by divergent evolution all known ACE1-containing coatomer systems (e.g. Y-Nups), their variants (e.g. IFTs) as well as a number of previously uncharacterized proteins (e.g. WDR17) is described by a very limited number of residues, predicted to play a role in the stability of this structural motif [54,55]. Despite highly integrated structural interpetations of the NPC [56] and transport vesicles [6], recent functional studies continue to unveil unexpected cellular roles for individual components and their interactions, e.g. in tRNA transcription [57] or pH regulation [58], respectively. Furthermore, the puzzling interplay between the NPC and the CPC to form a diffusion barrier [27,59] can now be illuminated by an evolutionary relationship of shared structural motifs. Parallel advances in the structural characterization of IFT proteins [60] and their interactions [61] might uncover the structure of the detected domains in some of the longest IFT proteins, e.g. IFT140 and IFT172. The biochemical basis of ciliary function in connection to signalling [62] coupled with novel structural insights might resolve the role of specific mutations in various ciliopathies [30,63].

References

47. Hughson, F.M. Copy coats: COPI mimics clathrin and COPII. *Cell* **142**, 19-21 (2010).
48. Devos, D.P., Graf, R. & Field, M.C. Evolution of the nucleus. *Curr Opin Cell Biol* **28C**, 8-15 (2014).
49. Onischenko, E. & Weis, K. Nuclear pore complex-a coat specifically tailored for the nuclear envelope. *Curr Opin Cell Biol* **23**, 293-301 (2011).
50. Holm, L. & Sander, C. An evolutionary treasure: unification of a broad set of amidohydrolases related to urease. *Proteins* **28**, 72-82 (1997).
51. Stohr, H. et al. Cloning and characterization of WDR17, a novel WD repeat-containing gene on chromosome 4q34. *Biochim Biophys Acta* **1579**, 18-25 (2002).
52. Alber, F. et al. The molecular architecture of the nuclear pore complex. *Nature* **450**, 695-701 (2007).
53. Brohawn, S.G. & Schwartz, T.U. Molecular architecture of the Nup84-Nup145C-Sec13 edge element in the nuclear pore complex lattice. *Nat Struct Mol Biol* **16**, 1173-7 (2009).
54. Leksa, N.C. & Schwartz, T.U. Membrane-coating lattice scaffolds in the nuclear pore and vesicle coats: commonalities, differences, challenges. *Nucleus* **1**, 314-8 (2010).
55. Miller, E.A. & Schekman, R. COPII - a flexible vesicle formation system. *Curr Opin Cell Biol* **25**, 420-7 (2013).
56. Bui, K.H. et al. Integrated structural analysis of the human nuclear pore complex scaffold. *Cell* **155**, 1233-43 (2013).
57. Ikegami, K. & Lieb, J.D. Integral nuclear pore proteins bind to Pol III-transcribed genes and are required for Pol III transcript processing in *C. elegans*. *Mol Cell* **51**, 840-9 (2013).
58. Kozik, P. et al. A human genome-wide screen for regulators of clathrin-coated vesicle formation reveals an unexpected role for the V-ATPase. *Nat Cell Biol* **15**, 50-60 (2013).
59. Obado, S.O. & Rout, M.P. Ciliary and nuclear transport: different places, similar routes? *Dev Cell* **22**, 693-4 (2012).
60. Bhogaraju, S., Taschner, M., Morawetz, M., Basquin, C. & Lorentzen, E. Crystal structure of the intraflagellar transport complex 25/27. *EMBO J* **30**, 1907-18 (2011).
61. Bhogaraju, S., Engel, B.D. & Lorentzen, E. Intraflagellar transport complex structure and cargo interactions. *Cilia* **2**, 10 (2013).
62. Delling, M., DeCaen, P.G., Doerner, J.F., Febvay, S. & Clapham, D.E. Primary cilia are specialized calcium signalling organelles. *Nature* **504**, 311-4 (2013).
63. Reiter, J.F., Blacque, O.E. & Leroux, M.R. The base of the cilium: roles for transition fibres and the transition zone in ciliary formation, maintenance and compartmentalization. *EMBO Rep* **13**, 608-18 (2012).

Supplementary Figures

Figure S1: Statistical measures of the sequence space walk. On the x-axis the thirteen steps are shown; on the left y-axis the number of entries corresponding to new hits (blue line) and the cumulative sum of hits (green line). Estimates for precision (red line) and recall (green line, corresponding to percentage points) are shown on the right y-axis (for actual values, see Table S2). While precision is never below 99%, coverage is slowly climbing from approximately 10% at step 1 to over 85% at step 13 – the total number of available sequence entries containing the ACE1-like motif is tentatively estimated at 4000.

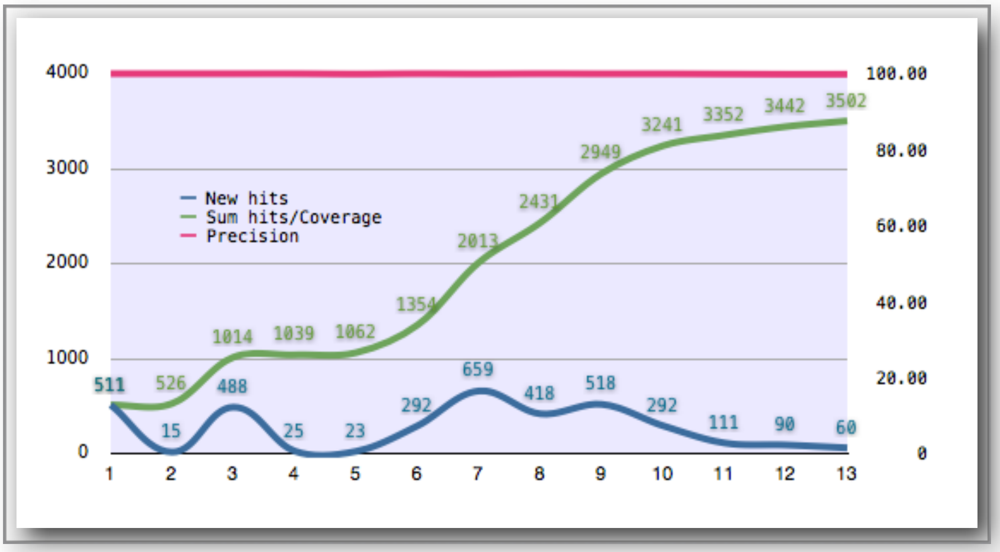

Figure S2: Global superposition of five available ACE1-like alpha-solenoid motif-containing structures. Viewpoint is maintained according to Figure 2; color scheme as in Figure 1. Despite the complexity of the five superimposed motifs, it is evident that they all exhibit a high degree of structural similarity, with the exception of Sec31's last helix hairpin, at the C-terminal region. Note that this structural superposition is purely sequence-driven with conserved residues (see main text for details).

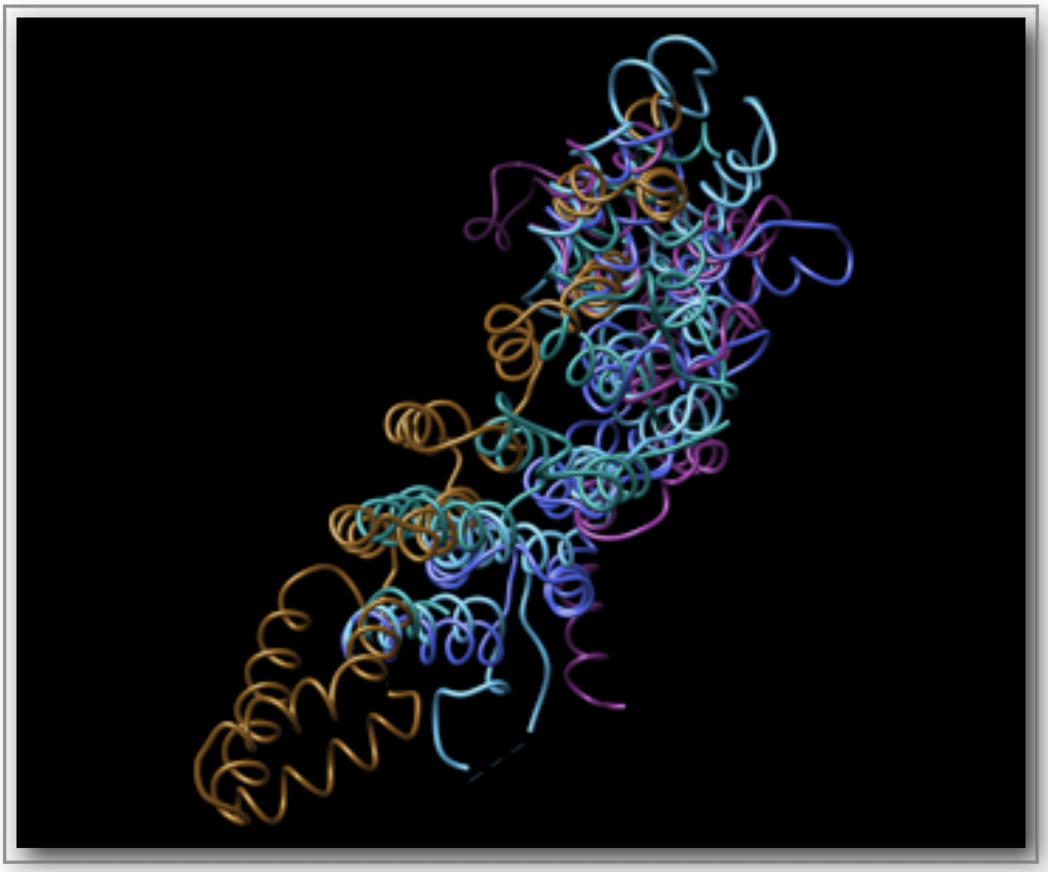

Figure S3: Index of the five eukaryotic coatomer structures with superfamily labels below. Viewpoint is maintained according to Figure 1; color scheme as in Figure 1. The purpose of this representation is solely to assist visualization of the complex global superposition in Figure S2.

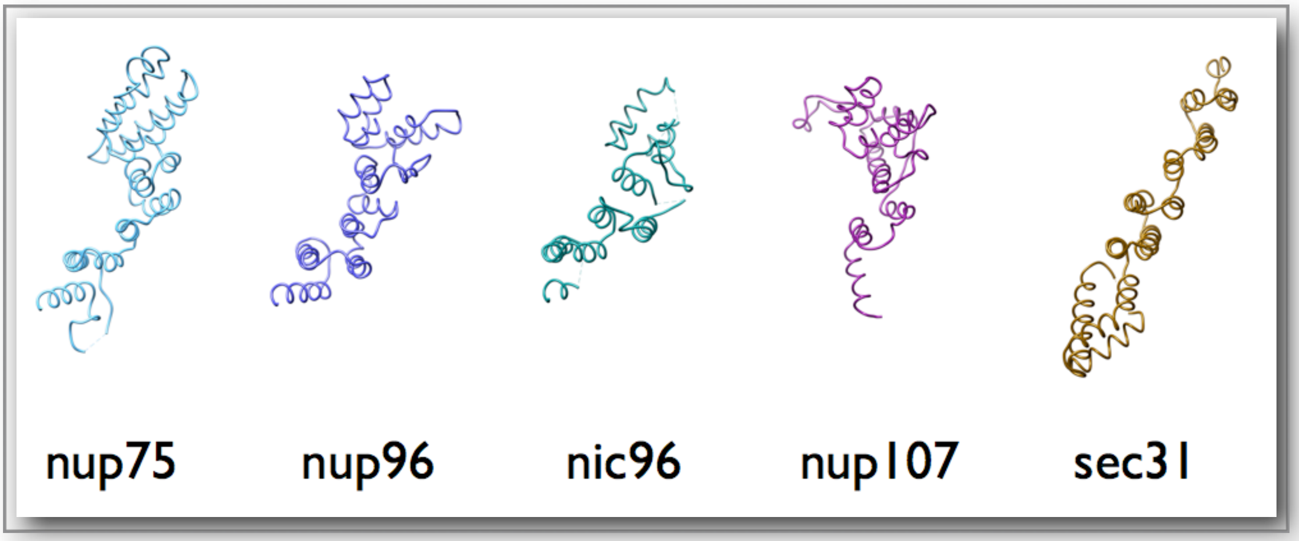

Supplementary Video

Video S1: A video representation of the sequence space walk capturing endomembrane coatomer superfamilies with increasing sensitivity. Top panel: a heat-map representation (yellow color corresponding to low values, blue color to high values) is shown for successive KMAP tables for all thirteen steps: on the x-axis, the twenty residue types are displayed and on the y-axis, the full sequence alignment positions – for display purposes only. Bottom panel: a sequence of screenshots for Figure 1 is shown where the limelight marks the relevant step, and the corresponding structure when available. Values change because the search does not converge as the profile is typically enriched at each step, notably for those steps where high numbers of related sequences are admitted.

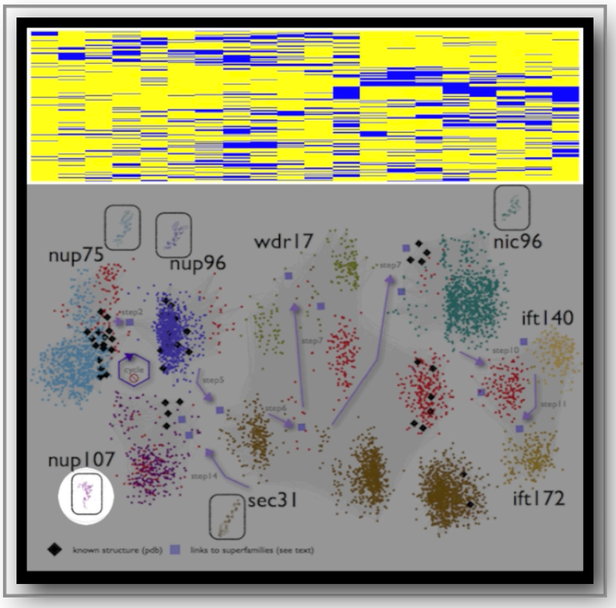

Supplementary Tables

Table S1

Distribution of putative false positive cases excluded from sequence profile constructions across the 13 steps described.

Step	Unique	Total	List of identifiers	Sum
1	0	0	none	0
2	3	3	XP_002784641.1 • XP_005537468.1 • XP_002771041.1	3
3	0	0	none	3
4	0	3	XP_002771041.1 • XP_002784641.1 • XP_005537468.1	3
5	1	1	XP_001015138.2	4
6	0	0	none	4
7	1	1	XP_002532671.1	5
8	0	0	none	5
9	0	1	XP_001015138.2	5
10	1	1	CDJ37712.1§	6
11	2	3	CDJ37712.1 • CDJ63560.1§ • CDJ51094.1	8
12	2	5	XP_002666781.2 • XP_004631041.1 • CDJ37712.1 • CDJ63560.1 • CDJ51094.1	10
13	5	5	XP_001846505.1 • EHH56549.1 • EHH23212.1 • XP_002598452.1 • ELW65852.1	15
	15	23		15

Column name, explanations: Step – corresponding sequence profile search; Unique – sum of unique false positives; Total – sum of all false positives including duplicates; List of identifiers – list of false positive identifiers; Sum – cumulative sum of unique false positives across steps. § Mark signifies possible true positive, unconfirmed (Sec31 homolog), does not impact search. Last row contains grand sums. Gray-colored labels signify identifiers which have been observed more than once.

Table S2

Description of 13 steps of profile sequence searches and relevant statistics.

Step	New	Total	PDB ids	min s.i.%	Cov	Pre	Comment
1	511	511	3f3fc 3eweb 3f3pc	9	12.78	100.00	mostly Nup75 members
2	15	526		8	13.15	100.00	first Nup96 from insects
3	488	1014		8	25.35	100.00	Nup75 and Nup96 connect
4	25	1039		8	25.98	100.00	few additional Nup96 hits
5	23	1062		8	26.55	99.91	first ACE1-containing protein
6	292	1354		8	33.85	100.00	Nup96 hits, first few Sec31A's
7	659	2013	2rfoa 2qx5a	7	50.33	99.95	Sec31's, few Nic96/WDR17's
8	418	2431	3ikob 3bg0b 3jroa	8	60.78	100.00	WDR17's, Nup107?, IFT140
9	518	2949		6	73.73	99.97	Nic96, IFT140, Sec31's
10	292	3241		6	81.03	99.97	above families, IFT140
11	111	3352		5	83.80	99.91	above families, first IFT172
12	90	3442		4	86.05	99.85	IFT140/IFT172, above families
13	60	3502	2pm6a	3	87.55	99.86	IFT140/IFT172, above families

Column name, explanations: Step – corresponding sequence profile search; New – New hits detected in the search step; Total – Cumulative sum of hits, including previous step; PDB ids – Four-character PDB identifiers and chain identifier (fifth character) detected in corresponding step; Min s.i.% – minimum sequence identity percent; Cov – coverage, estimated as total/4000; Pre – precision, estimated as total/(total+F^+); Comment – as in text (see Supplementary Text). Nup107 PDB identifiers not listed, as they are admitted only in the final step of the search, Step14. Counts (New, Total) and estimates (Cov, Pre) are shown in Figure S1.

Supplementary Data

Data Supplements DS01-DS04

The Nup75 sequence alignment, constructed from a redundancy-reduced alignment at 92% identity as in our previous study (Data Supplement DS01), is given in a trimmed form (Data Supplement DS02).

The resulting sequence profile is also provided in matrix (Data Supplement DS03) and ASN.1 (Data Supplement DS04) formats.

Data Supplement DS05

Sequence profile searches, 13 iterations, data output from PSI-BLAST, various formats: search strategy in ASN.1 format, hit table in comma-separated values (CSV) format, alignment in ASN.1 format, alignment in text format, alignment in XML format, alignment in tab-separated format, position-specific sequence matrix (PSSM) in ASN.1 format.

Data Supplement DS06

Keyword-extracted sequences ~5000 with Entrez® query.

Data Supplement DS07

Profile KMAP-13 (euKaryotic endoMembrane ACE1 Profile 13).

Data Supplement DS08

BLAST hit table for PSI-BLAST run with KMAP-13.

Data Supplement DS09

Pruned PDB file coordinates corresponding to sequence alignment (in Figure 2b).

Data Supplement DS10

DALI results.

Data Supplement DS11

Clustering validation. Text-based (refers to Data Supplement DS06) and sequence-based (refers to Data Supplement DS08) database entries. Representative nodes, for visualization purposes (with similarities at intervals -1/-5/-9/% including available PDB structures): 5983/6418=93% node coverage. Network has 101864 links, out of ~3M total. Corresponds to Figure 1.

Data availability

All results (in 11 Data Supplements) are available as a ZIP archive (117 MBytes) on http://dx.doi.org/10.6084/m9.figshare.1593170
